# Genetic relatedness of *Vibrio cholerae* isolates within and between households during outbreaks in Dhaka, Bangladesh

**DOI:** 10.1186/s12864-017-4254-9

**Published:** 2017-11-25

**Authors:** Christine Marie George, Mahamud Rashid, Mathieu Almeida, K. M. Saif-Ur-Rahman, Shirajum Monira, Md. Sazzadul Islam Bhuyian, Khaled Hasan, Toslim T. Mahmud, Shan Li, Jessica Brubaker, Jamie Perin, Zillur Rahman, Munshi Mustafiz, David A. Sack, R. Bradley Sack, Munirul Alam, O. Colin Stine

**Affiliations:** 1 Department of International Health, Program in Global Disease Epidemiology and Control, Johns Hopkins Bloomberg School of Public Health, 615 N. Wolfe Street, Room E5535, Baltimore, MD 21205-2103 USA; 2 International Centre for Diarrhoeal Disease Research, Shaheed Tajuddin Ahmed Ave, Dhaka, 1213 Bangladesh; 3 University of Maryland School of Medicine, 655 W Baltimore S, Baltimore, MD 21201 USA; 4 Johns Hopkins School of Public Health, 615 N. Wolfe Street, Baltimore, MD 21205-2103 USA

**Keywords:** *Vibrio cholerae*, Bangladesh, Whole genome sequencing, Multilocus variable-number tandem-repeat analysis, Outbreak surveillance, Household transmission, Genetics

## Abstract

**Background:**

Household contacts of cholera patients have a 100 times higher risk of developing a cholera infection than the general population. To compare the genetic relatedness of clinical and water source *Vibrio cholerae* isolates from cholera patients’ households across three outbreaks, we analyzed these isolates using whole-genome-sequencing (WGS) and multilocus variable-number tandem-repeat analysis (MLVA).

**Results:**

The WGS analyses revealed that 80% of households had source water isolates that were more closely related to clinical isolates from the same household than to any other isolates. While in another 20% of households an isolate from a person was more closely related to clinical isolates from another household than to source water isolates from their own household. The mean pairwise differences in single nucleotide-variant (SNV) counts for isolates from the same household were significantly lower than those for different households (2.4 vs. 7.7 *p* < 0.0001), and isolates from the same outbreak had significantly fewer mean pairwise differences compared to isolates from different outbreaks (mean: 6.2 vs. 8.0, p < 0.0001). Based on MLVA in outbreak 1, we observed that the majority of households had clinical isolates with MLVA genotypes related to other clinical isolates and unrelated to water source isolates from the same household. While in outbreak 3, there were different MLVA genotypes between households, however within the majority of households, the clinical and water source isolates had the same MLVA genotypes. The beginning of outbreak 2 resembled outbreak 1 and the latter part resembled outbreak 3. We validated our use of MLVA by comparing it to WGS. Isolates with the identical MLVA genotype had significantly fewer mean pairwise SNV differences than those isolates with different MLVA genotypes (mean: 4.8 vs. 7.7, *p* < 0.0001). Furthermore, consistent with WGS results, the number of pairwise differences in the five MLVA loci for isolates within the same household was significantly lower than isolates from different households (mean: 1.6 vs. 3.0, p < 0.0001).

**Conclusion:**

These results suggest that transmission patterns for cholera are a combination of person-to-person and water-to-person cholera transmission with the proportions of the two modes varying within and between outbreaks.

**Electronic supplementary material:**

The online version of this article (10.1186/s12864-017-4254-9) contains supplementary material, which is available to authorized users.

## Background

The World Health Organization estimates that there are 3–5 million cholera cases worldwide per year resulting in more than 100,000 deaths [1]. Studies have identified water [2–5] and food borne contamination [6, 7] to be the main transmission routes for cholera. Household contacts are at a 100 times higher risk of developing a cholera infection than the general population [3, 8–10]. However, most previous studies among this high risk population were conducted before genetic identification of *Vibrio cholerae* strains was available.

Genetic identification of *V. cholerae* strains allows for sources of infection in a household to be identified. One genetic method, whole genome sequencing (WGS) distinguishes between isolates based on single nucleotide variants (SNVs). WGS data has revealed three phylogenetically distinct waves of cholera spreading around the world [11] and has been shown to be useful in outbreak investigation to identify separate outbreaks within a single time period [12]. A second genetic method, multilocus variable-number tandem-repeat analysis (MLVA) distinguishes between different strains of *V. cholerae* based on the number of short (6 to 9), repeating nucleotide sequences at five loci. An observational study of household contacts of cholera cases using MLVA in Dhaka, Bangladesh found that *V. cholerae* strains were genetically identical at five loci between index cases and household contacts for only 46% of pairs analyzed [13]. This result is very different than the nearly 90% matching by serogroup and serotype.

A recent study compared MLVA and WGS and found that they reflected the same genetic history [14], in contrast to two earlier reports [15, 16]. Rashid et al. found that isolates closely related by MLVA had significantly fewer nucleotides differences when compared to each other than when compared to isolates distantly related by MLVA [14]. In this study Rashid et al. reported about a shorter time scale (less than 1 year vs 38 years) than the first report [16] and did more extensive sampling than the second report [15].

We recently conducted a randomized controlled trial of a health facility based handwashing with soap and water treatment intervention for the household contacts of cholera patients (Cholera-Hospital-Based Intervention-for-7-Days (CHoBI7) Trial) to reduce cholera among this high risk population in Dhaka, Bangladesh [17]. In an attempt to investigate transmission patterns within cholera-patient households, we performed pulsed-field gel electrophoresis (PFGE) on clinical and water isolates collected from patient households in this trial. Of the 33 *V. cholerae* isolates analyzed by PFGE, 88% were found to have identical banding patterns [18]. The close similarity between clinical and water isolates within patient households is suggestive of the household’s drinking water being the source of infecting inoculum in these homes.

Building on this previous work in our current study, we will compare the genetic relatedness of *V. cholerae* O1 isolates from cholera patients, their household members, and their household water sources by WGS and MLVA. These methods will allow us to differentiate between *V. cholerae* O1 isolates that are typically indistinguishable by PFGE [13, 19, 20]. Our objective is to investigate cholera transmission patterns in patient households over a 1 year period to determine if all outbreaks are the same or if there is variability in transmission patterns across outbreaks.

## Results

### Epidemiology

**Fig. 1:**
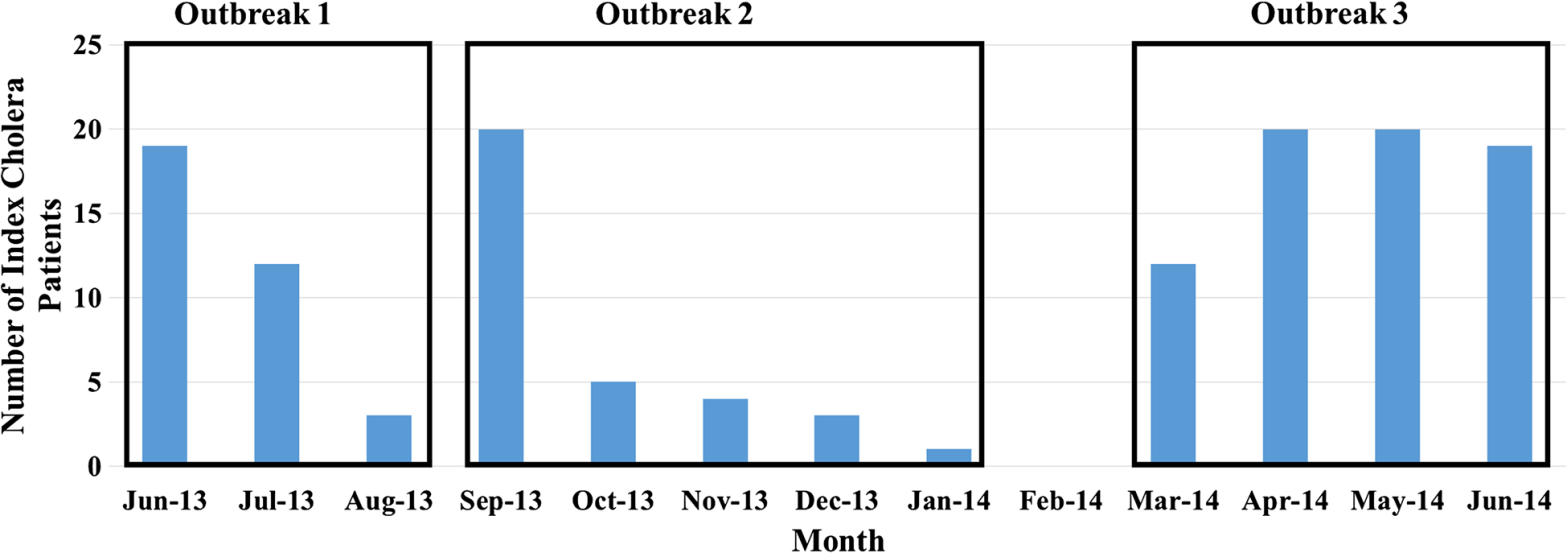
The number of cholera patients included in clinical surveillance during three successive outbreaks at icddr,b Dhaka Hospital, June 2013 to June 2014

**Fig. 2:**
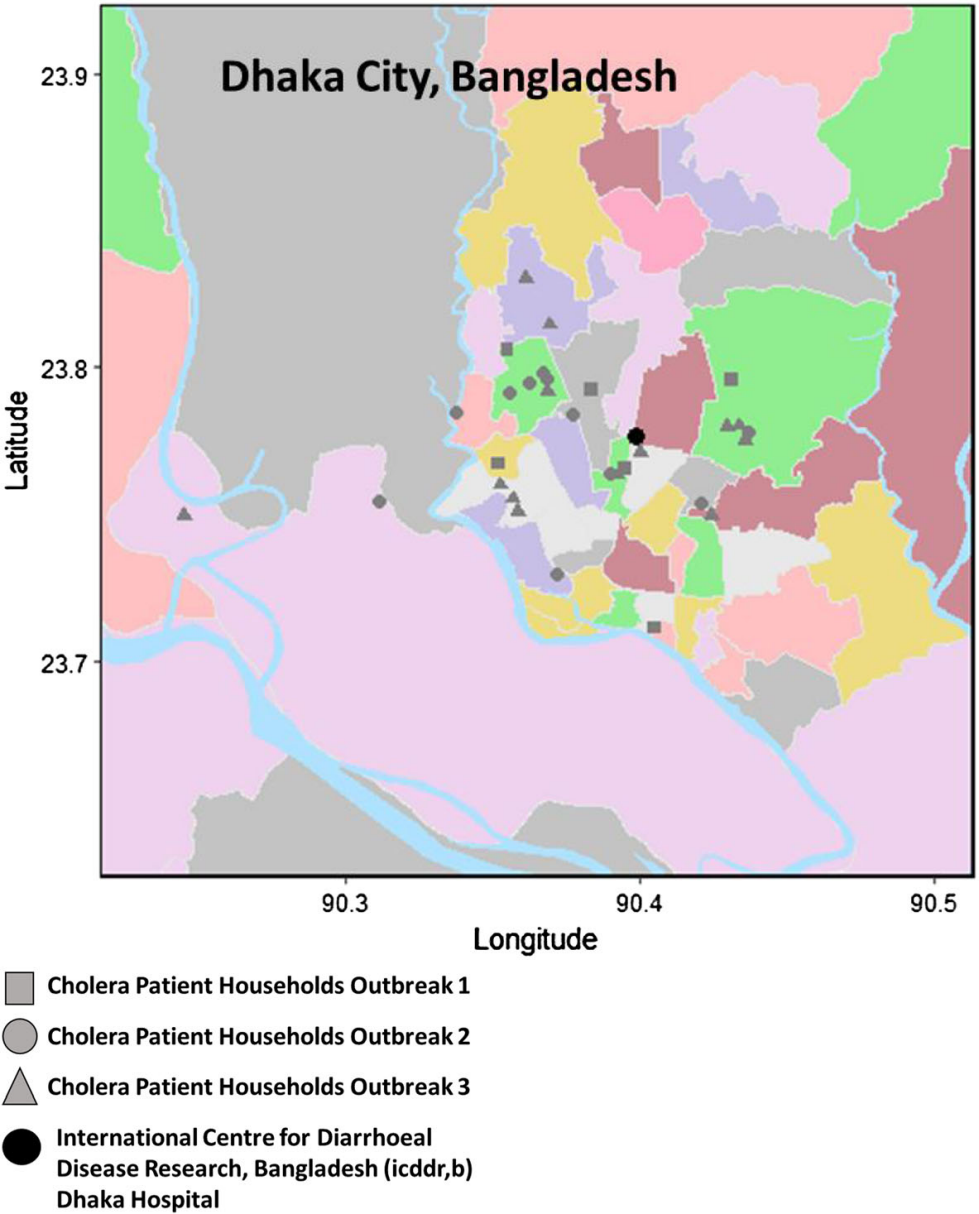
Map of Cholera Patient Households in Dhaka City. The black circle is the International Centre for Diarrhoeal Disease Research, Bangladesh (icddr,b) Dhaka Hospital. Squares are cholera patient households from Outbreak 1, circles are cholera patient households from Outbreak 2, and triangles are cholera patient households from Outbreak 3. Thana (ward) boundaries for Dhaka City were defined using the Humanitarian Data Exchange (https://data.humdata.org/)

Our 1 year surveillance period identified a total of 136 culture confirmed cholera patients with three distinct case based outbreaks which were separated by a month with less than 4 cholera patients (Fig. 1). There was a summer peak (June –August 2013) with 33 cholera patients, a fall peak (September 2013 –January 2014) with 33 cholera patients, and a spring peak (March to June 2014) with 70 cholera patients. During these outbreaks, 19% of household contacts and 30% of drinking water sources used in cholera-patient households had detectable *V. cholerae* O1 by culture*.* A total of 621 isolates, 288 clinical and 333 water *V. cholerae* O1 isolates were analyzed from 31 households in which both patient and water samples were positive, 27 households had a patient and *source* water samples available. For each positive sample within the household, up to 10 *V. cholerae* O1 Ogawa isolates were collected. There was no significant difference in the location of cholera-patient households across outbreaks (*p* = 0.251) (Fig. 2). Average distances between households were 6.5, 5.4, and 6.8 km in outbreaks 1, 2, and 3, respectively. In addition, there was no apparent clustering of the households based on the outbreak.

### Whole genome sequencing

Thirty-eight genomes of O1 isolates collected from 17 households were sequenced, 13 households had multiple isolates, and 10 had both water and clinical isolates (Additional file 1: Table S1). Thirteen genomes were from Outbreak 1, 12 from Outbreak 2, and 13 from Outbreak 3. On average, each genome was assembled into 80.9 scaffolds (Additional file 2: Table S2), with an average depth of 415 reads. After aligning the genomes as described in the methods section, we identified 66 SNVs distributed in 29 different scaffolds, with 81% of them corresponding to the chromosome I and mostly in regulatory genes.

**Fig. 3:**
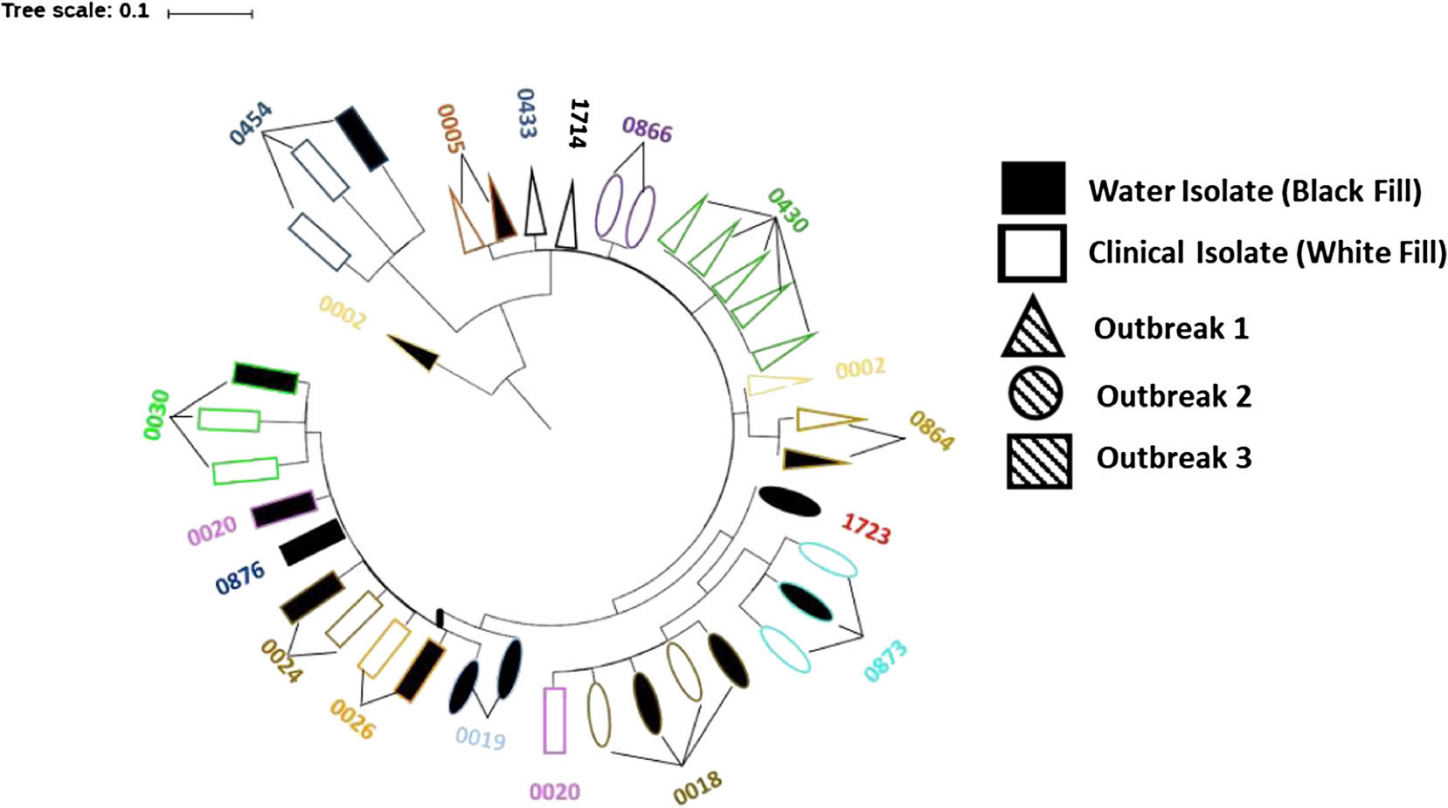
Maximum likelihood phylogram of the genetic relatedness of selected isolates based on WGS. Isolates are represented by shapes: isolates from Outbreak 1 are triangles, Outbreak 2 are ovals, and Outbreak 3 are rectangles. Black filled shapes are isolates from water and white filled shapes are from clinical isolates. Color outline on shapes represents households. Shapes with the same outline color are from the same household. Black color outline is for four isolates where only one strain was sequenced in the household. The numbers are the household IDs. The length of the radial lines connecting isolates are proportional to number of SNVs between isolates. The SNVs were identified using the strain S002604 as the reference for the core-genome alignment and using only the contigs bigger than 10Kb to remove the potentially low assembly quality regions, which represent less than 5% of the genomes

**Fig. 4:**
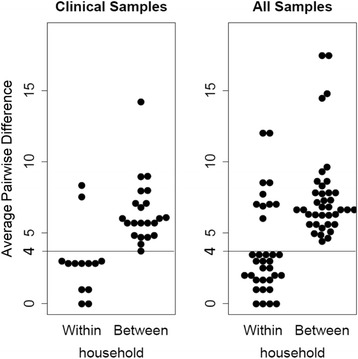
Average Pairwise SNV differences within and between households for **a**) clinical samples from people and **b**) all samples

We estimated the genetic relatedness of the 38 genomes using a maximum likelihood tree calculated from the variants detected in the alignments of the scaffolds bigger than 10 kb (Fig. 3). Only high quality SNVs were used in the bee swarm plot (Fig. 4). The mean pairwise differences in SNV counts for isolates from the same households (mean: 2.4 (range: 0–12)) were significantly lower than those for different households (7.7 (range: 1–21)) (*p* < 0.0001). Within the household, clinical isolates averaged less than or equal to 4 SNVs differences, while between households the average difference is greater than 4. The same is true when isolates from water sources are added to the comparison, isolates within the household average less than or equal to 4 SNVs differences, while for isolates between households the average difference is greater than 4. If an average of four differences is accepted as a threshold for determining within versus between household transmission of *V. cholerae*, then two households out of 10 households with clinical and source water isolates (20%) (0002 & 0020, see Fig. 3) had a person acquire their infecting *V. cholerae* outside the home or a source we did not measure. While for the other 8 out of 10 households with clinical and water isolates (80%), the genotype of the source water isolate in the household was more closely related to that of infected individuals in the same household than any other isolate.

The differences between groups of isolates are shown in Table 1. Source water isolates had significantly greater mean pairwise differences compared to clinical isolates (mean: 9.0 vs. 6.2, *p* < 0.0001). Isolates from the same outbreak had significantly fewer mean pairwise differences compared to isolates from different outbreaks (mean: 6.2 vs. 8.0, p < 0.0001). When stratified by isolate type (clinical vs. water), isolates from the same outbreak had significantly fewer pairwise differences for clinical isolates (*p* < 0.0001), but not for water isolates (*p* = 0.26).

**Table 1:** Pairwise comparisons of single nucleotide variant (SNV) counts for *Vibrio cholerae* clinical and water isolates by WGS

	Pairwise Comparisons*	All Outbreaks	*p*-value†
		Mean SNV ± SD (Min-Max)	
Inter vs. Intra Household Variability in SNV Counts			
Isolates from Same Households	33	2.4 ± 3.1 (0–12)	<0.0001
Isolates from Different Households	670	7.7 ± 4.8 (1–21)	
Same MLVA Genotype vs. Different MLVA Genotype Variability in SNV Counts			
Same MLVA Genotype	77	4.8 ± 4.9 (0–18)	<0.0001
Different MLVA Genotype	628	7.7 ± 4.8 (0–21)	
Single Locus Variants in MLVA Genotype vs. Different MLVA Genotype Variability in SNV Counts			
Single Locus Variants in MLVA Genotypes	89	4.7 ± 3.1 (0–15)	<0.0001
Different MLVA Genotype	539	8.2 ± 4.8 (1–21)	
Clinical vs. Water Isolate Variability in SNV Counts			
Clinical Isolates	231	6.2 ± 3.7 (1–18)	<0.0001
Water Isolates	120	9.0 ± 6.1 (1–20)	
Same vs. Different Outbreaks Variability in SNV Counts			
Isolates from Same Outbreak	232	6.1 ± 4.9 (0–21)	0.01
Isolates from Different Outbreak	473	8.0 ± 4.8 (1–21)	

†Permutation Tests

*Comparisons of Pairs of Isolates

*SD* Standard Deviation

### Multilocus variable-number tandem-repeat analysis

Substantial genetic variation in the collected isolates was observed by MLVA. The genotype of each isolate was specified by the alleles at the five loci. There were 8 alleles at VC0147, 10 at VC0437, 9 at VC1650, 11 at VCA0171, and 9 at VCA0283. One hundred twenty-four distinct genotypes were identified: 25 genotypes from clinical isolates, 81 from water isolates, and 18 genotypes that were found in both clinical and water isolates.

Multiple MLVA genotypes were observed in isolates collected from a single clinical or water sample. The variation within a single sample was greater for water isolates (mean: 6 genotypes, range: 2–10 genotypes) compared to clinical isolate (mean: 2 genotypes, range: 1–5) (*p* < 0.0001). Three quarters (76%, 42/55) of clinical samples and all (100%, 46/46) of water samples had at least two isolates with different MLVA genotypes (Additional file 1: Table S1). For clinical specimens, the proportion of specimens with more than one MLVA genotype increased significantly over the course of the three outbreaks from 47% to 100% (p < 0.0001), no significant difference was observed for water samples (Table 2). Of note, one allele at VCA0171 did not amplify from 99 DNA sequences despite repeated attempts; subsequent WGS revealed the locus is present, but a mutation altered the last nucleotide of the primer binding site and interfered with amplification. Thirty percent (99/333) of water isolates had an unamplified allele at VCA0171 compared to none of clinical isolates (0/288) (p < 0.0001). The unamplified allele was present in 66% of the water isolates in the first outbreak and in only 1% of the water isolates in the third outbreak (p < 0.0001).

**Table 2:** Relatedness of clinical and water MLVA genotypes of *Vibrio cholerae* by outbreak

	All Outbreaks	Cholera Outbreaks			*p*-value†
		Outbreak 1	Outbreak 2	Outbreak 3	
		June to August 2013	September 2013 to January 2014	March to June 2014	
Samples with at least two isolates with different MLVA genotypes					
Clinical Samples	76% (42/55)	47% (8/17)	79% (15/19)	100% (19/19)	<0.0001
Water Samples	100% (46/46)	100% (8/8)	100% (15/15)	100% (23/23)	‡
Isolates with unamplified VCA0171 Allele					
Clinical Isolates	0% (0/288)	0% (0/139)	0% (0/109)	0% (0/40)	‡
Water Isolates	30% (99/333)	66% (52/79)	39% (46/118)	1% (1/136)	<0.0001
Household Characteristics					
Household member and water isolates with identical MLVA genotypes	58% (18/31)	50% (3/6)	55% (6/11)	64% (9/14)	0.09
Index cholera patient and *source water* isolate with identical MLVA genotypes	56% (15/27)	40%(2/5)	56% (5/9)	62% (8/13)	0.09
Household member and *source water isolates* with identical MLVA genotypes	52% (16/31)	50% (3/6)	46% (5/11)	57% (8/14)	0.09
Household member and *stored water* isolates with identical MLVA genotypes	40% (2/5)	0% (0/1)	50% (1/2)	50% (1/2)	0.4
Household member isolates with identical MLVA genotypes	82% (9/11)	66% (2/3)	75% (3/4)	100% (4/4)	0.2

†Fisher Exact Test

‡ Fisher exact couldn’t be calculated because all proportions were the same across outbreaks

The majority of households had clinical and water isolates with identical MLVA genotypes. Eighty-two percent (9/11) of households had infected household members with identical MLVA genotypes. In 56% (15/27) of households, an isolate from the index case had the identical genotype as a *source* water isolate. In 58% (18/31) of households, there were infected household members *and* water isolates (stored and source water isolates) with identical MLVA genotypes (Table 2). Conversely, in 42% of households, there were no identical genotypes found in both household member and water samples.

**Fig. 5:**
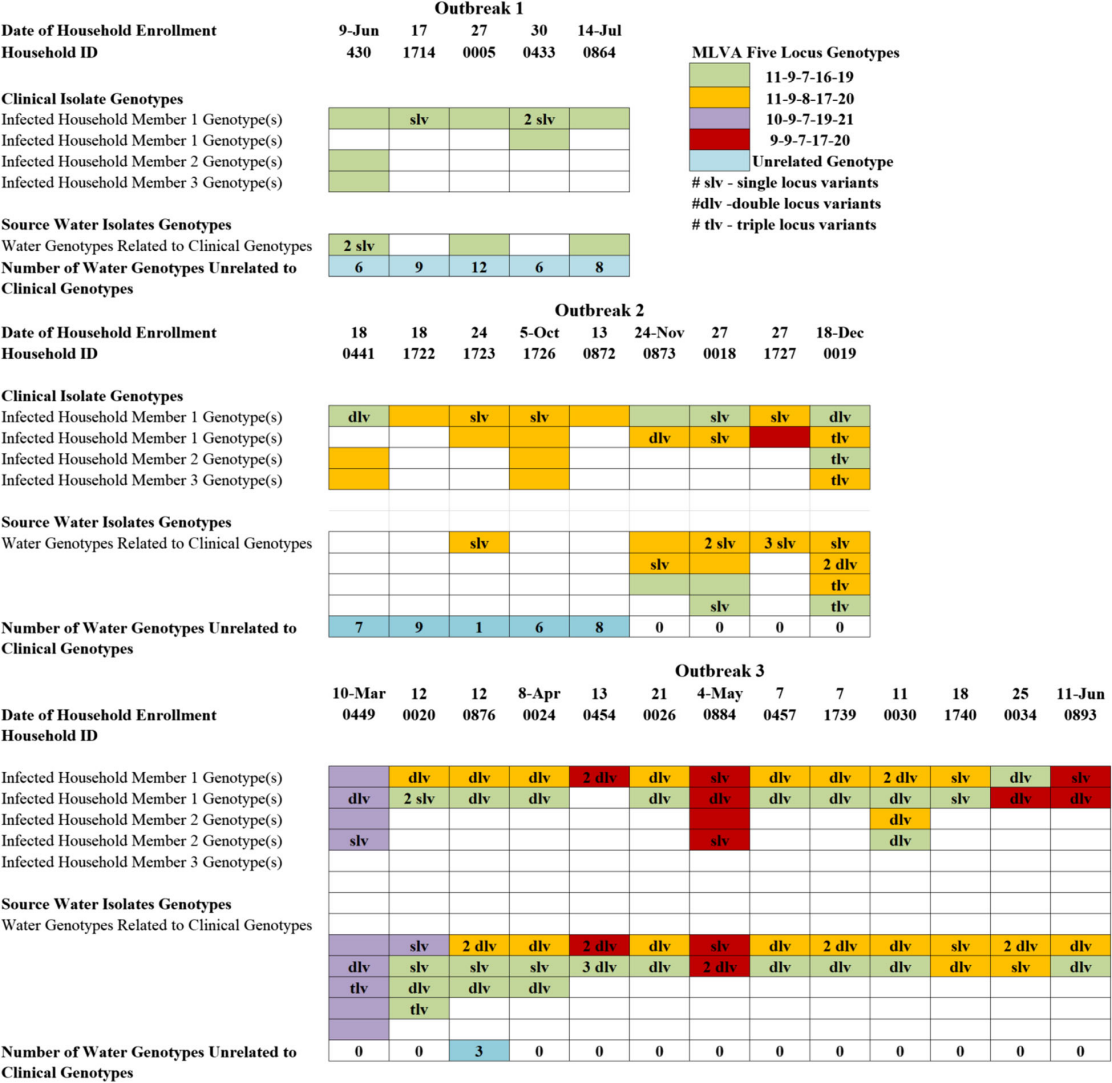
Genetic relatedness of isolates from clinical and water samples across three outbreaks. Each section represents one outbreak. Within a section, the dates of household enrollment, and household IDs are shown. In each column, the genetic lineage is identified by color. Boxes without letters indicate a specific genotype: green = 11–9–7-16-19, orange = 11–9–8-17-20, purple = 10–9–7-19-21, and red = 9–9–7-17-20. The letters slv, dlv and tlv represent single, double and triple locus variants. The numbers represent the number of genotypes. The blue boxes identify genotypes that are unrelated to the clinical genotypes in the same household

The genetic relatedness of *V. cholerae* isolates in patients and water differed substantially between the three outbreaks (Fig. 5). In the first outbreak, there was a single predominate MLVA genotype found in 93% (13 of 14) of clinical specimens and the other seven clinical MLVA genotypes in this outbreak were single-locus variants (SLVs) of this genotype. In contrast, of the 44 MLVA genotypes found in the water isolates during this outbreak, only 3 were the same or SLVs of the genotype in infected individuals within these households. In the second outbreak, there were two MLVA genotype lineages found in the infected household members. One was the MLVA lineage from the first outbreak observed in nine clinical specimens and six households. The second MLVA lineage was observed in twelve clinical samples and nine households. Of note, while only one of these two lineages was in any given individual prior to November 2013, after that date four of six infected household members had both lineages represented. There was a similar finding for water samples, up until November 2013 for Outbreak 2 there were 31 MLVA genotypes that were unrelated to the isolates in the clinical specimens. After November, all of the isolates observed in the water were related to the two predominate MLVA lineages. In the first household of Outbreak 3, a new MLVA lineage was observed in both patients and in the water, it also was found in the water of the second household. However the second and subsequent households look like the second portion of the second outbreak. The water from Outbreak 3 households contained the two previous lineages from outbreak 2 in ten households, a third lineage in two other households, and a fourth in three more households.

### Validation of MLVA by WGS

Isolates with eighteen different MLVA genotypes were analyzed by WGS. Isolates with the identical MLVA genotype had significantly fewer mean pairwise SNV differences than those isolates with different MLVA genotypes (mean: 4.8 vs. 7.7, *p* < 0.0001). Furthermore, isolates that differed at a single MLVA locus were more closely related than those with greater MLVA allelic variation (mean: 4.70 vs. 8.23, *p* < 0.001).

Consistent with results from WGS, isolates collected within households were more closely related by MLVA than those from different households. The number of pairwise differences in the five MLVA loci for isolates within a household was significantly lower than isolates from different households (mean: 1.6 vs. 3.0, p < 0.0001). The lower number of pairwise differences was significant even when stratified by clinical (mean: 0.29 vs. 2.1, p < 0.0001) and water isolates (mean 1.7 vs. 3.3, p < 0.0001). Water isolates also had a significantly greater number of MLVA loci differences compared to clinical isolates (mean: 3.3 vs. 2.0, p < 0.0001). Isolates from the same outbreak had significantly fewer MLVA loci differences compared to isolates from different outbreaks (mean: 2.5 vs. 3.2, p < 0.0001).

## Discussion

Our results are consistent with the presence of two modes of cholera transmission: person-to-person and water-to-person with the proportions of the two modes varying within and between outbreaks. Our WGS analyses of 38 isolates revealed that 80% of households had source water isolates that were more closely related to clinical isolates from the same household than to any other isolates. While in another 20% of households, an isolate from a person was more closely related to clinical isolates from another household than to source water isolates from their own household. We interpret the former to be water-to-person transmission and the latter to be person-to-person transmission. In order to expand the sample size, we used MLVA to estimate the genetic relatedness of 621 systematically collected isolates. Our estimates of relatedness by MLVA reflected the relatedness that was observed by WGS. Based on our MLVA, we observed differences in transmission patterns between and within outbreaks. In Outbreak 1, we observed that the majority of households had clinical isolates with MLVA genotypes unrelated to water source isolates while clinical isolates within households were all genetically related, consistent with person-to-person transmission. In contrast, in Outbreak 3, there were different MLVA genotypes between households, although within the household, clinical and water source isolates had the same MLVA genotypes consistent with water-to-person transmission. Outbreak 2 showed a combination of both modes of transmission with the beginning of the outbreak resembling Outbreak 1 and the latter part resembling Outbreak 3. Therefore, these findings indicate that in this urban setting cholera control programs should focus on both hygiene promotion, to reduce person-to-person transmission, and on municipal and point of use water treatment programs, to reduce water-to-person transmission.

The genotypes from isolates within a household were more closely related than those between households. We therefore suspect the spread of cholera within many households comes from a contaminated water source used for drinking water by household members, as evidenced by the WGS and MLVA data showing that water and clinical isolates within a household are more closely related than isolates outside the household. Alternatively, there were instances when the spread of cholera within the household came from other infected household members through poor hygiene practices, as evidenced in Outbreak 1 where household members had the same MLVA genotypes, while the water source isolates for the most part were unrelated to the clinical isolates.

We observed more water than clinical MLVA genotypes, while in previous studies, more clinical genotypes were observed than water genotypes [21, 20]. This is likely the result of study design and sampling issues with previous studies. In each of the previous studies, Rashed et al. [21] and Stine et al. [20] a single colony was picked from each water sample likely resulting in an underestimation of the variation in the water. When comparing only clinical samples, Kendall et al. [13] in another household contact study of cholera patients found 83 distinct MLVA clinical genotypes. However this study was conducted over a time span of more than 3 years compared to our 1 year period, perhaps allowing more time to accumulate additional clinical MLVA genotypes. In addition, our results revealed the majority of those infected with cholera had multiple MLVA genotypes present in their stool samples consistent with previous work [13, 21].

In our study, WGS and MLVA data reflected similar genetic relationships for comparisons made between *V. cholerae* isolates. Isolates with the same MLVA genotype were significantly more similar by WGS than isolates with different MLVA genotypes. In addition, isolates that differed at a single MLVA locus were also significantly more similar than those that differed at more than one MLVA locus. This finding is consistent with previous work by Rashid et al. who showed that distinct MLVA clonal complexes represent separate WGS lineages [14].

Our study has several strengths. The first was the environmental surveillance of the source and stored drinking water in the households of cholera patients. Second, we conducted WGS and MLVA which allowed us to complement our WGS analysis with a larger number of strains analyzed by MLVA. Third, we collected multiple isolates from all clinical and water samples which allowed us to investigate diversity of MLVA genotypes within samples.

Our study has a few limitations. First, the 621 water and clinical isolates collected were limited to 31 households spread over 3 outbreaks. Second, we only included households that had a water sample with detectable *V. cholerae*. Third, we only sequenced a fraction of the isolates we collected, although given the high correlation between MLVA and WGS changes in the conclusions seem unlikely.

## Conclusion

These results provide evidence consistent with two modes of cholera transmission, water-to-person and person-to-person transmission, within and between the households of cholera patients during outbreaks in Dhaka, Bangladesh. Remarkably, person-to-person transmission was the dominate mode of transmission in one outbreak while water-to-person transmission was the dominate mode in subsequent outbreaks.

## Methods

The 31 households from the CHoBI7 randomized controlled trial (RCT) [17] in Dhaka, Bangladesh included in this analysis were enrolled from June 2013 to June 2014. Cholera patients confirmed by bacterial culture presenting at the icddr,b Dhaka hospital Sunday to Thursday were enrolled. A description of the intervention is described elsewhere [17]. Case households were visited at Days 1, 3, 5, 7, and 9 after the index patient was identified. At each visit, rectal swab samples were collected from household contacts and a water sample was collected from the household’s water source, a piped connection to the municipal water supply, and stored drinking water in the home to test for *V* *. Cholerae* by bacterial culture. Household contacts were defined as individuals sharing the same cooking pot with the cholera patient for the past 3 days.

### Sample collection and processing

Stool samples from the index patient were collected at the hospital. Rectal swab samples from household contacts and water samples were collected from the households of enrolled cholera patients. All samples were processed and analyzed by bacterial culture and serotyped using published methods [17, 22]. For clinical samples, up to 5 colonies were selected and for each water sample up to 10 colonies.

### Whole genome sequencing (WGS)

Genomic DNA was extracted from 38 isolates using published phenol-chloroform extraction methods [23]. Genome sequencing was performed as previously published on an Illumina HiSeq2500 (Illumina, San Diego, CA, USA) [24, 25].

High quality reads of the 101-base paired-end reads were selected (https://www.bioinformatics.babraham.ac.uk/projects/fastqc/), assembled with “Spades” software (v.3.6.2) [26], and annotated using the RAST server [27]. The assembled genomes were submitted to Genbank and are associated with the BioprojectID: PRJNA371610. We choose the wave 3 isolate genome, S002604, as the reference, in order to minimize the number of variable sites that were different between the reference genome and all the genomes in our sample since those variable sites do not contribute to the analysis. PARSNP (v1.2) [28] was used to extract and align the variable nucleotides using previously published options [24, 25]. The ‘.ggr’ file was loaded in Gingr (v1.2) [28] to visualize the alignments. A ‘.vcf’ file was used to remove all variants less than 1 kb from the end of the contigs in the ‘.mfa’ file using an in-house script. FastTree2 (v2.1.9) [29] generated the maximum-likelihood newick tree file using the revised alignment file. iTOL (http://itol.embl.de/) [30] was employed to visualize the maximum-likelihood tree.

### Multilocus variable-number tandem-repeat analysis (MLVA)

To complement the WGS analysis with a larger number of strains, MLVA was performed on DNA from 621 *V. cholerae* water and clinical isolates from patient households. DNA was isolated from 5 μl of culture using Prepman (ABI) according to the manufacturer’s instructions. To perform MLVA, the DNA from the *V*. *cholerae* O1 isolates was genotyped at each of five previously identified MLVA loci (VC0147, VC0437, VC1650, VCA0171 & VCA0283) using previously published methods [13].

### Statistical analysis

Fisher’s exact, paired t-tests, permutation tests, and Poisson regression models were computed using SAS (version 9.3) to analyze WGS and MLVA data. For WGS, pairwise comparisons were made of the SNV counts for each isolate compared to the reference strain, S002604. For MLVA, the relatedness of *V. cholerae* isolates was assessed using all five MLVA loci. Pairwise comparisons were made based on the number of loci with different alleles (e.g. there would be one difference if a single locus varied, two if two loci varied). The statistical analysis of the cholera patient household locations was performed using R version 3.3.2 with package ggplot.

## Additional files

Additional file 1: Table S1. Genome Metadata. (XLSX 22 kb)

Additional file 2: Table S2. SNP List of Chromosomes and Genes using reference 300,043. (XLSX 19 kb)

## Abbreviations

icddr,b: International Centre for Diarrhoeal Disease Research, Bangladesh
MLVA: Multilocus variable-number tandem-repeat analysis
PFGE: Pulsed-field gel electrophoresis
SLVs: Single-locus variants
SNVs: Single nucleotide variants
WGS: Whole genome sequencing

## References

[1] Organization WH. WHO | Cholera - World Health Organization. http://www.who.int/cholera/en/.

[2] Deb B, Sircar B, Sengupta P, De S, Mondal S, Gupta D, Saha N, Ghosh S, Mitra U, Pal S (1986). Studies on interventions to prevent eltor cholera transmission in urban slums. Bull World Health Organ.

[3] Hughes JM, Boyce JM, Levine RJ, Khan M, Aziz K, Huq M, Curlin GT (1982). Epidemiology of eltor cholera in rural Bangladesh: importance of surface water in transmission. Bull World Health Organ.

[4] Kumar P, Mishra DK, Deshmukh DG, Jain M, Zade AM, Ingole KV, Goel AK, Yadava PK (2014). Vibrio cholerae O1 Ogawa el tor strains with the ctxB7 allele driving cholera outbreaks in south-western India in 2012. Infect Genet Evol.

[5] Bhuyan SK, Vairale MG, Arya N, Yadav P, Veer V, Singh L, Yadava PK, Kumar P (2016). Molecular epidemiology of Vibrio cholerae associated with flood in Brahamputra River valley, Assam, India. Infect Genet Evol.

[6] Sinclair G, Mphahlele M, Duvenhage H, Nichol R, Whitehorn A, Küstner H (1982). Determination of the mode of transmission of cholera in Lebowa. An epidemiological investigation. S Afr Med J.

[7] Acosta CJ, Galindo CM, Kimario J, Senkoro K, Urassa H, Casals C, Corachán M, Eseko N, Tanner M, Mshinda H (2001). Cholera outbreak in southern Tanzania: risk factors and patterns of transmission. Emerg Infect Dis.

[8] Weil AA, Khan AI, Chowdhury F, LaRocque RC, Faruque A, Ryan ET, Calderwood SB, Qadri F, Harris JB (2009). Clinical outcomes in household contacts of patients with cholera in Bangladesh. Clin Infect Dis.

[9] Spira W, Khan MU, Saeed Y, Sattar M (1980). Microbiological surveillance of intra-neighbourhood el tor cholera transmission in rural Bangaldesh. Bull World Health Organ.

[10] Glass RI, Svennerholm AM, Khan MR, Huda S, Huq MI, Holmgren J (1985). Seroepidemiological studies of el tor cholera in Bangladesh: association of serum antibody levels with protection. J Infect Dis.

[11] Mutreja A, Kim DW, Thomson NR, Connor TR, Lee JH, Kariuki S, Croucher NJ, Choi SY, Harris SR, Lebens M (2011). Evidence for several waves of global transmission in the seventh cholera pandemic. Nature.

[12] Shah MA, Mutreja A, Thomson N, Baker S, Parkhill J, Dougan G, Bokhari H, Wren BW (2014). Genomic epidemiology of Vibrio cholerae O1 associated with floods, Pakistan, 2010. Emerg Infect Dis.

[13] Kendall EA, Chowdhury F, Begum Y, Khan AI, Li S, Thierer JH, Bailey J, Kreisel K, Tacket CO, LaRocque RC (2010). Relatedness of Vibrio cholerae O1/O139 isolates from patients and their household contacts, determined by multilocus variable-number tandem-repeat analysis. J Bacteriol.

[14] Rashid MU, Almeida M, Azman AS, Lindsay BR, Sack DA, Colwell RR, Huq A, Morris JG Jr, Alam M, Stine OC. Comparison of inferred relatedness based on multilocus variable-number tandem-repeat analysis and whole genome sequencing of Vibrio cholerae O1. FEMS Microbiol Lett. 2016;363(12) 10.1093/femsle/fnw116 PMC4876684 27190166

[15] Abd El Ghany M, Chander J, Mutreja A, Rashid M, Hill-Cawthorne GA, Ali S, Naeem R, Thomson NR, Dougan G, Pain A (2014). The population structure of Vibrio cholerae from the Chandigarh Region of Northern India. PLoS Negl Trop Dis.

[16] Lam C, Octavia S, Reeves P, Wang L, Lan R (2010). Evolution of seventh cholera pandemic and origin of 1991 epidemic, Latin America. Emerg Infect Dis.

[17] George CM, Monira S, Sack DA, Rashid MU, Saif-Ur-Rahman KM, Mahmud T, Rahman Z, Mustafiz M, Bhuyian SI, Winch PJ (2016). Randomized controlled trial of hospital-based hygiene and water treatment intervention (CHoBI7) to reduce cholera. Emerg Infect Dis.

[18] Rafique R, Rashid MU, Monira S, Rahman Z, Mahmud MT, Mustafiz M, Saif-Ur-Rahman KM, Johura FT, Islam S, Parvin T (2016). Transmission of infectious Vibrio cholerae through drinking water among the household contacts of cholera patients (CHoBI7 trial). Front Microbiol.

[19] Danin-Poleg Y, Cohen LA, Gancz H, Broza YY, Goldshmidt H, Malul E, Valinsky L, Lerner L, Broza M, Kashi Y (2007). Vibrio cholerae strain typing and phylogeny study based on simple sequence repeats. J Clin Microbiol.

[20] Stine OC, Alam M, Tang L, Nair GB, Siddique AK, Faruque SM, Huq A, Colwell R, Sack RB, Morris JG (2008). Seasonal cholera from multiple small outbreaks, rural Bangladesh. Emerg Infect Dis.

[21] Rashed SM, Azman AS, Alam M, Li S, Sack DA, Morris JG, Longini I, Siddique AK, Iqbal A, Huq A (2014). Genetic variation of Vibrio cholerae during outbreaks, Bangladesh, 2010-2011. Emerg Infect Dis.

[22] Alam M, Sultana M, Nair GB, Siddique AK, Hasan NA, Sack RB, Sack DA, Ahmed KU, Sadique A, Watanabe H (2007). Viable but nonculturable Vibrio cholerae O1 in biofilms in the aquatic environment and their role in cholera transmission. Proc Natl Acad Sci U S A.

[23] Chowdhury NR, Chakraborty S, Ramamurthy T, Nishibuchi M, Yamasaki S, Takeda Y, Nair GB (2000). Molecular evidence of clonal Vibrio Parahaemolyticus pandemic strains. Emerg Infect Dis.

[24] Kachwamba Y, Mohammed AA, Lukupulo H, Urio L, Majigo M, Mosha F, Matonya M, Kishimba R, Mghamba J, Lusekelo J (2017). Genetic characterization of Vibrio cholerae O1 isolates from outbreaks between 2011 and 2015 in Tanzania. BMC Infect Dis.

[25] Garrine M, Mandomando I, Vubil D, Nhampossa T, Acacio S, Li S, Paulson JN, Almeida M, Domman D, Thomson NR (2017). Minimal genetic change in Vibrio cholerae in Mozambique over time: multilocus variable number tandem repeat analysis and whole genome sequencing. PLoS Negl Trop Dis.

[26] Bankevich A, Nurk S, Antipov D, Gurevich AA, Dvorkin M, Kulikov AS, Lesin VM, Nikolenko SI, Pham S, Prjibelski AD (2012). SPAdes: a new genome assembly algorithm and its applications to single-cell sequencing. J Comput Biol.

[27] Overbeek R, Olson R, Pusch GD, Olsen GJ, Davis JJ, Disz T, Edwards RA, Gerdes S, Parrello B, Shukla M (2014). The SEED and the rapid annotation of microbial genomes using subsystems technology (RAST). Nucleic Acids Res.

[28] Treangen TJ, Ondov BD, Koren S, Phillippy AM (2014). The harvest suite for rapid core-genome alignment and visualization of thousands of intraspecific microbial genomes. Genome Biol.

[29] Price MN, Dehal PS, Arkin AP (2010). FastTree 2--approximately maximum-likelihood trees for large alignments. PLoS One.

[30] Letunic I, Bork P (2016). Interactive tree of life (iTOL) v3: an online tool for the display and annotation of phylogenetic and other trees. Nucleic Acids Res.

